# Antioxidant Extracts of Nettle (*Urtica dioica*) Leaves: Evaluation of Extraction Techniques and Solvents

**DOI:** 10.3390/molecules27186015

**Published:** 2022-09-15

**Authors:** María Flórez, Patricia Cazón, Manuel Vázquez

**Affiliations:** Department of Analytical Chemistry, Faculty of Veterinary, University of Santiago de Compostela, 27002 Lugo, Spain

**Keywords:** *Urtica dioica*, antioxidant properties, plant extracts, DPPH^•^, ABTS^•+^

## Abstract

Nettle (*Urtica dioica*) is a great source of bioactive compounds. The objective of this study was to evaluate the extraction techniques (ultrasound, without stirring, and stirring), solvents (methanol, water, and ethanol), and extraction times (1–4 h) to maximize antioxidant capacity of the *Urtica dioica* extracts. In the case of total phenolic content (TPC) and ABTS^•+^ (2,2-azino-bis(3-etilbenzotiazolin)-6-sulfonic acid) free radical scavenging values, ultrasound extraction was the most efficient method, while the best results of DPPH^•^ (1,1-diphenyl-2-picrylhydrazyl) assay in nettle extracts (91.08%) were obtained using stirring extraction, water as solvent, and 3 h of extraction time. Based on the obtained mathematical models, the optimization revealed that the best extraction conditions were ultrasound treatment with water as solvent and an extraction time of 3.15 h, obtaining values of 21.9 mg eq gallic acid/g dried nettle for TPC, 71.8% for %ABTS^•+^ and 86.6% for %DPPH^•^. This work proves that aqueous extract of nettle leaves through the ultrasound technique is an important source of natural antioxidants and can be considered a potential alternative to synthetic antioxidants.

## 1. Introduction

Plants are a great source of natural ingredients and active compounds. Over the centuries, herbs and plants have been widely applied in foods as seasonings and preservatives. They improve the sensory characteristics of foods and help prolong their shelf-life by reducing or eliminating pathogenic bacteria. The natural components of plants could meet the demands of consumers who are asking for food products with minimal processing, less use of synthetic additives and without disturbing food safety.

The use of natural extracts from herbs and plants could replace some synthetic additives. Natural extracts are a great source of antioxidants and active compounds, such as polyphenols, flavonoids, etc., used in the food and packaging industries [1]. The parts of the plant most commonly used for extraction are the leaves [2], fruits [3], seeds [4], or roots [5]. Most of these plant extracts have a high content of phenolic compounds that provide antioxidant properties, thus preserving food by reducing lipid oxidation [6]. For this reason, they can be an alternative to synthetic antioxidants.

The antioxidant plant extracts can be used in active packaging. This term refers to the design of biodegradable polymers mixed with active compounds, such as natural extracts, which provide antimicrobial and/or antioxidant properties. The active compound added to the matrix deliberately interacts with the food, thus extending its shelf-life. In this way, plant extracts have received a lot of interest because they contain a high concentration of phenolic components, which have strong antioxidant properties. The incorporation of these plant extracts into packaging is a promising strategy for preventing or reducing food quality deterioration, therefore improving food preservation and shelf life extension [7]. Black corn extract, pomegranate peel extract, or *Pistacia terebinthus* extract are just a few examples of natural extracts that can be added to active packaging [8]. It is important to note that the use of these extracts in polymers produces changes in mechanical, barrier, antioxidant, antimicrobial, and physicochemical properties that must be assessed. 

The Urticaceae family is considered the main group of Angiosperms (flowering plants), and within it is the genus *Urtica*. *Urtica dioica*, commonly known as nettle, is a perennial plant. This plant is composed of a stem that can reach 10–50 cm in height and that holds up its characteristic rough and serrated leaves [9]. 

Among the main components of nettle leaves are polyphenolic compounds, such as flavonoids and phenolic acids (syringic, caffeic, ferulic, gallic acids). These compounds are recognized for their antioxidant, anti-inflammatory, antiviral capacity, among others. It has been reported that both flavonoids and phenolic acids carry out their antioxidant activity through various mechanisms. The predominant mechanism is believed to be radical scavenging through hydrogen atom donation [10]. On the other hand, chlorophylls, carotenoids, and fatty acids are also important compounds in the composition of nettle. The chlorophyll and carotenoids play a role in nettle as light-harvesting pigments. Specifically, carotenoids can suppress harmful photochemical reactions involving oxygen, therefore antioxidant capacity is attributed to them. Fatty acids are also present in the nettles, being the unsaturated fatty acids the most abundant. The predominant fatty acids are *α*-linolenic acid, palmitic acid, and *cis*-9,12-linoleic acid. The fatty acids n-3 and n-6 are found in significant proportions, providing anti-inflammatory, vasodilatory, antithrombotic, and hypolipidemic properties [11].

The nettle leaves are characterized by the content of Na, K, Ca, Mg, Fe, and Mn. Three elements (Pb, Cd, and As) considered contaminants were found in trace concentrations. It should be taken into account that the composition of the nettle varies according to the time of harvesting, the soil on which it has grown, the exposure of the plant to the sun, and other factors [12]. 

The extraction process of natural products can be carried out through several steps: (I) The solid matrix is penetrated by the solvent, (II) the solute obtained is dissolved in the solvent, (III) the solute is removed from the solid matrix, (IV) the extracted solutes are collected. Several methods can be used for extraction. 

Traditional procedures, such as Soxhlet extraction, reflux, and maceration extraction take a long time, consume a lot of solvent, and may cause thermolabile chemicals to degrade due to the higher temperatures utilized. In recent years, other techniques have begun to be used, such as ultrasound-assisted extraction. This process allows greater penetration by the solvent into the materials to be extracted, improving mass transfer thanks to the effects of the micro-streaming [13]. Therefore, this technique is considered an efficient extraction process that has benefits, such as reduced time, increased yields, or low temperature. It often improves the quality of the extract. 

On the other hand, stirring extraction is a method where the sample is kept stirred in a controlled environment, which allows for better permeability of the solvent in the matrix. Oppositely, the without stirring extraction refers to the immersion of the plant matter in a solvent by controlling only the extraction temperature [14]. 

Plant matter, extraction procedures, and solvents affect the efficiency of the extraction of bioactive components from plants. Solubility and diffusion of extract increase at high temperatures. However, temperatures that are excessively high might cause the destruction of thermolabile components and solvent evaporation, resulting in undesirable impurities in the extract. On the other hand, the extraction efficiency improves as the extraction time increases. Nevertheless, when the solute has reached equilibrium, increasing the extraction time will have no effect on the efficiency of the process. The particle size of the plant material is also an important feature since the efficiency of the extraction will be enhanced by the small size of the particles to better penetrate the solvents and facilitate the diffusion of the solutes [15]. Moreover, polar solvents, such as methanol, water, or ethanol are commonly used to obtain plant extracts. Therefore, the choice of the extraction technique and solvent is critical for bioactive component extraction efficiency [16]. 

The purpose of this work was to assess the extraction process for obtaining the maximum antioxidant activity in extracts obtained from dried nettle *Urtica dioica* leaves. The technique (ultrasound, without stirring, and stirring extraction), solvent (methanol, ethanol, or water), and extraction time (1 to 4 h) were evaluated. Mathematical models were obtained to predict the best operational conditions, and the antioxidant capacity of dried nettle extracts was optimized.

## 2. Results and Discussion

Nettle leaves were dried before use for the antioxidant extraction. Moisture of dried nettle leaves was very low (0.60 ± 0.02)%. A moisture percentage of less than 12% is considered of good quality and adequate for the analysis [12]. The dried nettle leaves showed a water activity value of 0.264. This value was very low and ensured that the raw material was very stable [17]. 

The drying process decreases the water activity of the samples, which inhibits microbial growth and minimizes the biochemical reactions that lead to spoilage [18]. Pathogenic bacteria cannot grow below a water activity of 0.85–0.86. Taking this into account, it is interesting that the nettle extracts have such low values of water activity (0.264), in order to combat microbial growth [17], and guarantee the storage stability of dried nettles over time. Moreover, the drying process of the plant could modify the microstructure of the plant tissues that make up the nettle. This also could produce a better extraction yield [19]. 

**Figure 1:**
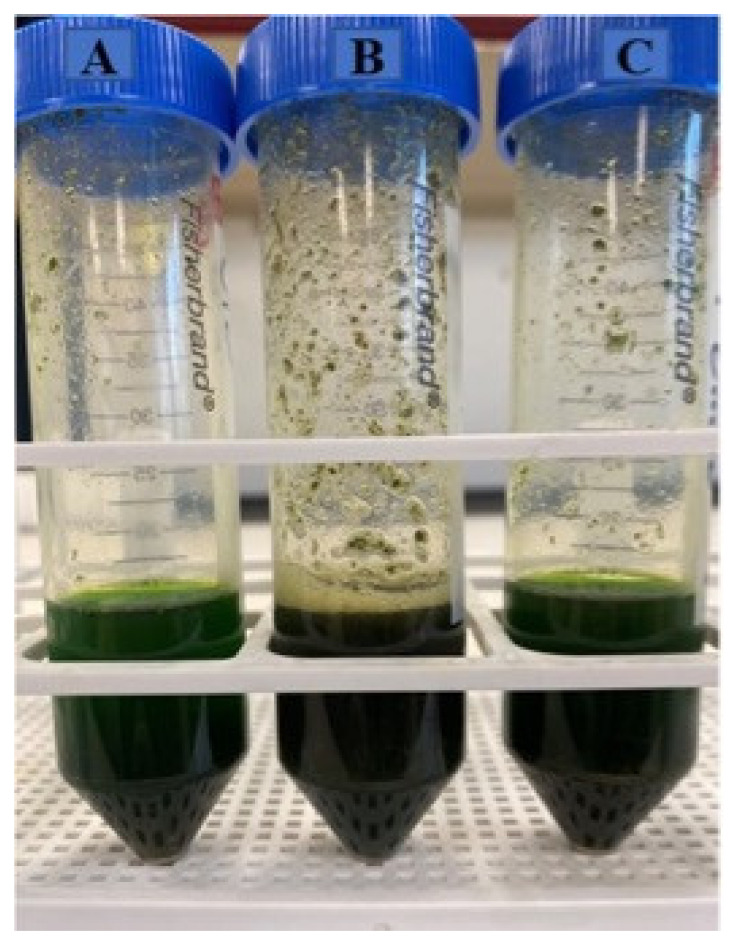
Samples of methanolic (A), aqueous (B) and ethanolic (C) extracts from nettle leaves.

Table 1 shows the experimental design with the 36 experiments carried out and the antioxidant values obtained measured in triplicate. The type of solvent influenced the color of the extracts. The extracts of dried nettle leaves obtained using methanol and ethanol as solvent showed a strong green color, meanwhile the aqueous extract had a brownish color (Figure 1).

The experimental results were used to obtain mathematical models to predict the best operational conditions. The analysis of variance (ANOVA) presented in Table 2 shows that all the models obtained are statistically significant. Table 2 also shows the fit statistics, whereas the predictive models for the antioxidant methods can be seen in Table 3.

### 2.1. Modelling of the Total Phenolic Content (TPC)

The total phenolic content (TPC) values ranged from 3.3 to 27.0 mg eq gallic acid/g dried nettle (Table 1). Data fitted well to a two-factor interaction mathematical model. The ANOVA indicated that the obtained mathematical model was significative (*p* < 0.05). The TPC of dried nettle samples depended on the time, extraction method, and solvent used. The F-value and *p*-value of the model were 20.85 and 2.14 × 10^−9^, respectively. The *p*-values of the model terms indicated that the lineal effect of time (A), method (B), and solvent (C) and the interaction between time-method (AB), time-solvent (AC), and method-solvent (BC) were significant (*p* < 0.05) (Table 2). The F-values of the terms can be used to determine which component of the model had the greatest effect on the response. The effect of solvent used (F-value = 80.10) and the extraction time (F-value = 20.56) were those that showed the greatest effect on the extraction of phenolic compounds of the nettle samples. 

The fit statistics results indicated a r^2^ of the predictive model of 0.92. The adjusted r^2^ is used to compare goodness-of-fit for regression models with different numbers of independent variables. On the other hand, the predicted r^2^ assesses how well the model predicts a response value. For reasonable agreement, the difference between the adjusted r^2^ and the predicted r^2^ should be below 0.20. In this case, the predicted r^2^ (0.81) is in reasonable agreement with the adjusted r^2^ (0.88). Adequate precision measures the signal-to-noise ratio, this is used to compare the range of predicted values at the design points against the mean error of the model prediction [20]. A model discrimination ratio of more than 4 shows adequate model discrimination. In this case, the adequate precision value was 20.28, which indicates an adequate signal (Table 2). 

Through the predictive equations obtained for each method (Table 3), it is possible to predict the TPC values in the range of the time studied, with the solvents and extraction methods used. 

**Figure 2:**
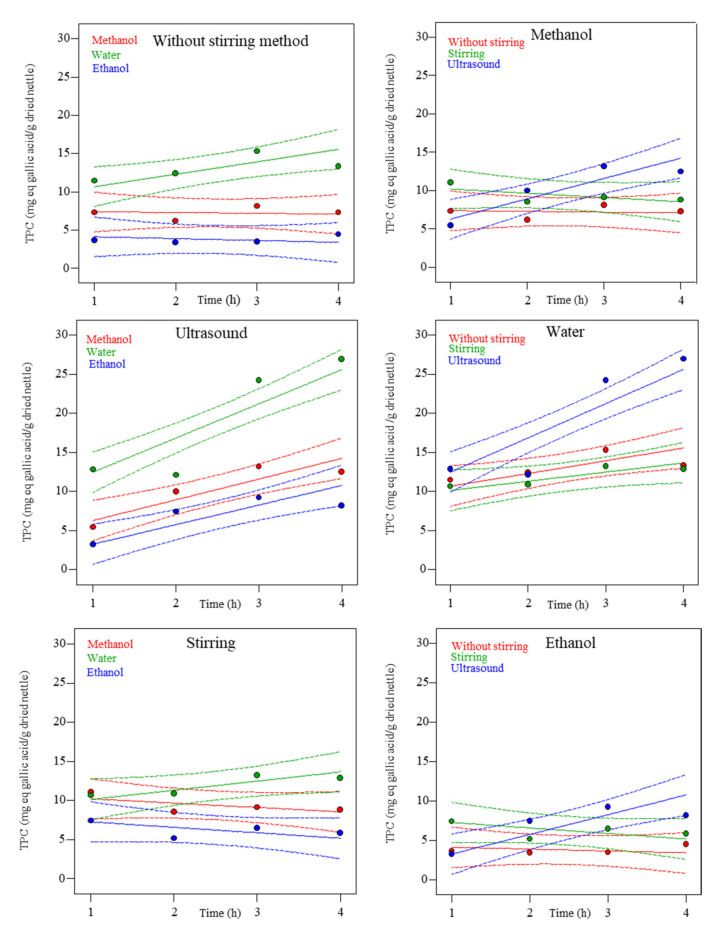
Experimental and predicted dependence of Total Phenolic Content (TPC) on time at different methods (without stirring, ultrasound, or stirring) and solvents (methanol, water, or ethanol). Dots are experimental results, dotted lines are 95% confidence bands, and solid lines are the predicted results.

Figure 2 shows data (colored dots) and the predicted values of the mathematical model (solid lines), depending on the solvent (red: Methanol, green: Water, and blue: Ethanol) and the extraction method (red: Without stirring, green: Stirring and blue: Ultrasound) for TPC values. The dotted lines refer to the 95% confidence bands.

The highest TPC values were achieved with the ultrasound extraction (27.0 mg eq gallic acid/g dried nettle). This could be explained by the fact that in the ultrasound process, the solvent produced by cavitation accelerated the dissolution and diffusion of the solute, which improved the extraction efficiency [15]. Similar results were obtained when ultrasound was used as the extraction method and water as solvent [21]. The second one was without stirring extraction and the worse was stirring extraction. 

On the other hand, the use of water as solvent showed the highest TPC values (from 10.7 to 27.0 mg eq gallic acid/g dried nettle) as well as producing a significant effect on the antioxidant capacity of the extracts. Methanol was the second better solvent to achieve the higher results (from 5.5 to 13.2 mg eq gallic acid/g dried nettle), while the extraction with ethanol generated the lowest TPC values in the final extracts (from 3.6 to 9.3 mg eq gallic acid/g dried nettle). 

According to data, the extract that achieves the best TPC results is obtained through the ultrasound method, with water as solvent and 4 h of extraction time. This can be explained because water is a good solvent for phenolic acids and glycosides that may be present in nettle. This generates higher extraction yields of these compounds than other organic solvents, such as ethanol or methanol [22]. The same behavior was observed [23] when they studied the total phenolic content of aromatic and medicinal plants with water as a solvent. 

Extraction time produced a significant effect on nettle extracts. However, in extractions with organic or hydroalcoholic solvents, no significant degradation of phenolic compounds has been reported at longer extraction times [24]. 

### 2.2. Modelling of DPPH^•^ Radical Scavenging Activity

The DPPH^•^ radical scavenging activity of the obtained extracts ranged from 25.9 to 91.1%. Note that the samples of extracts were subjected to a 1/100 dilution, expecting more potent antioxidant activity in pure extracts. Data fitted well to a quadratic mathematical model (Table 2). The model had an F-value of 7.30 and *p*-value of 3.07 × 10^−5^, indicating that it is significant (*p* < 0.05). Considering the *p*-values, all terms were significant except the linear effect of the extraction method (*p*-value of 0.3150) and the time-method interaction (*p*-value of 0.3291). The F-values showed that the solvent used (F-value = 25.55) and the time of extraction (F-value = 13.09) had a higher effect on the DPPH^•^ radical scavenging activity response. The fit statistics values indicated a r^2^ value of 0.83. The predicted r^2^ had a value of 0.44, and the adjusted r^2^ was 0.72, which indicated that these values were not as close as would be expected, and the difference was greater than 0.2. Nevertheless, the model had an adequate precision of 9.00, suggesting that it had an adequate signal-to-noise ratio. 

**Figure 3:**
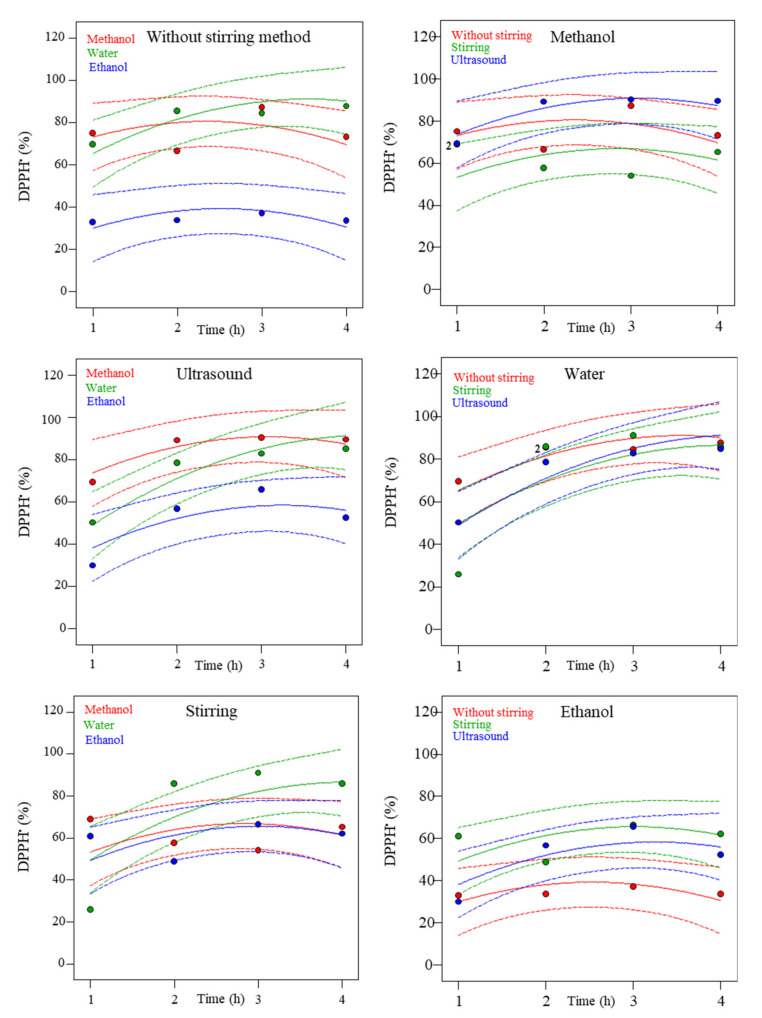
Experimental and predicted dependence of DPPH^•^ (1,1-Diphenyl-2-picrylhydrazyl) assay on time at different methods (without stirring, ultrasound, or stirring) and solvents (methanol, water, or ethanol). Dots are experimental results, dotted lines are 95% confidence bands, and solid lines are the predicted results.

Table 3 shows the mathematical models that forecast the DPPH^•^ values. Figure 3 shows experimental results (colored dots) and the predicted values of the mathematical model (solid lines), depending on the solvent (red: Methanol, green: Water and blue: Ethanol) and the extraction method (red: Without stirring, green: Stirring and blue: Ultrasound) for DPPH^•^ values. 

It was observed that different DPPH^•^ values were obtained depending on the solvent used for the three methods analyzed. For the without stirring method, the highest value was obtained using water (87.7%), while for the ultrasound method, the highest value was obtained using methanol (90.2%), and for the stirring extraction, the highest value was obtained using water (91.1%).

It can be seen the relationship between the method of extraction and the solvent used. Thus, water was the solvent that showed the highest DPPH^•^ values followed by methanol and ethanol. Hence, the aqueous extract subjected to any of the three extraction methods obtained high DPPH^•^ values in 3 h. This could be explained by the fact that the use of polar solvents showed higher affinity, allowing to obtain extracts with higher antioxidant potential than with slightly polar solvents [25]. Similar behavior was observed when antioxidant methods were studied on *Spirulina platensis*, obtaining values of DPPH^•^ of 95.3% with water as solvent after 30 min of incubation of the samples [26].

### 2.3. Modelling of ABTS^•+^ Radical Scavenging Activity

The values of the ABTS^•+^ radical scavenging activity of the 1/100 diluted extracts obtained from the dried nettles for each extraction method and solvent ranged from 8.1 to 91.8% (Table 1). Data fitted well to a two-factor interaction mathematical model. The F-value of the model was 24.85, and the *p*-value was 3.78 × 10^−10^, indicating that the mathematic model was significant (*p* < 0.05). Considering the *p*-values of the model terms, all terms were significant except for the time-solvent interaction (*p*-value of 0.1432). The fit statistics values were r^2^ of 0.94, predicted r^2^ of 0.82, and adjusted r^2^ of 0.90. In this case, the predicted r^2^ and the adjusted r^2^ were in reasonable agreement. The adequate precision (17.95) demonstrated that the model had an adequate signal. Table 3 shows the predictive equations for ABTS^•+^ values for each method and solvent used. 

**Figure 4:**
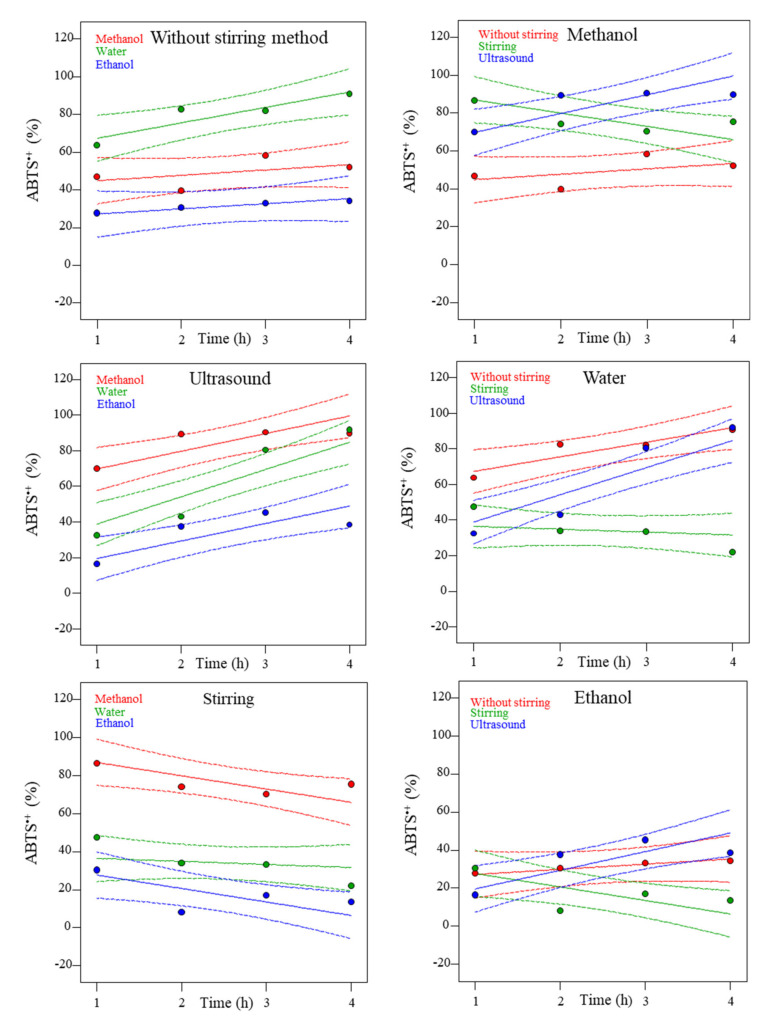
Experimental and predicted dependence of ABTS^•+^ (2,2-azino-bis(3-etilbenzotiazolin)-6-sulfonic acid) assay on time at different methods (without stirring, ultrasound, or stirring) and solvents (methanol, water, or ethanol). Dots are experimental results, dotted lines are 95% confidence bands, and solid lines are the predicted results.

Figure 4 shows the experimental results (colored dots) and the predicted values of the mathematical models (solid lines), depending on the solvent (red: Methanol, green: Water and blue: Ethanol) and the extraction method (red: Without stirring, green: Stirring, and blue: Ultrasound) for ABTS^•+^ values.

The samples subjected to ultrasound extraction using water as solvent showed better results. The method of extraction applied depended on the solvent used. For the ultrasound extraction, the highest ABTS^•+^ values were obtained (91.8%) using water for 4 h of time extraction. For without stirring conditions, the highest ABTS^•+^ values were obtained also using water for 4 h (90.8%). However, for the stirring method, the best solvent was methanol for 1 h (86.6%). 

On the other hand, the relationship between solvent used and method of extraction used as function of the time, showed that water promoted the highest extraction efficiency (from 21.84 to 91.83%). Methanolic extracts were the second best one (from 39.6 to 90.4%), while ethanol was the solvent with the lowest results (from 8.1 to 42.2%). Based on the results obtained, it can be assumed that ultrasound aqueous extraction is the best procedure. These results agree with the literature. The ultrasound method also gave better antioxidant values in mango seed extraction [27].

This behavior could be attributed to the fact that ABTS^•+^ assay is aqueous-based, hence hydrophilic phenolic compounds with antioxidant capacity, such as caffeic malic acid, are favored [28].

### 2.4. Optimization of the Antioxidant Compounds Extraction of Urtica dioica Nettles

Based on the obtained mathematical models, the optimization was carried out by establishing the priority criteria. The priority criterium was estimated on a scale from 1 (lowest priority) to 5 (highest priority), where each parameter and response assigned the following values: Time = 3, method = 3, solvent = 3, %ABTS^•+^ = 3, %DPPH ABTS^•^ = 3, and TPC = 5. According to the established criteria and the predictive models, the results of the process optimization indicated that the most suitable extraction would be using ultrasound treatment with water as solvent and an extraction time of 3.15 h. In this way, the predicted antioxidant capacity of the nettle extracts was a value of TPC of 21.9 mg eq gallic acid/g dried nettle, %ABTS^•+^ of 71.8%, and %DPPH^•^ of 86.6%.

## 3. Materials and Methods

### 3.1. Chemicals and Plant Materials

Folin–Ciocalteu reagent from Panreac (Barcelona, Spain). DPPH and ABTS were purchased from Alfa Aesar (Haverhill, MA, USA). Methanol, ethanol, and sodium carbonate were purchased from Scharlau Microbiology (Barcelona, Spain).

*Urtica dioica* was collected from Luarca (Asturias, Spain) from January to February 2022. In the laboratory, the plants were cleaned and defoliated to obtain healthy leaves of uniform color and size, and their stems and petioles were removed. The nettles’ leaves were dried in a dehydrator at 45 °C for 24 h (Excalibur^®^ Food dehydrator, Sacramento, CA, USA) to remove the water content. Afterwards, the leaves were crushed and milled in an electric mill to obtain powder and subsequently stored in airtight bottles at room temperature. 

### 3.2. Determination of Moisture Content of the Dry Matter and Water Activity (Aw)

The moisture content and the dry matter of the samples were determined. For this purpose, 1 g of sample was weighed and dried at 110 °C in an oven for 24 h. The percentage of moisture was calculated from the mass difference after and before drying. The water activity (a_w_) of the dried nettle powder was determined with a water activity measuring equipment, Aqualab^®^ (METER Group, Pullman, WA, USA). All the analyses were performed in triplicate.

### 3.3. Preparation of Extracts

Three methods were evaluated for extraction: Ultrasound-assisted extraction, without stirring extraction, and stirring extraction. In all cases, 1 g of dried nettle powder was weighed, transferred to a tube with a screw cap, and diluted with 15 mL of the solvent (methanol, ethanol, or water). Ultrasound-assisted extraction was performed at 65 °C in an ultrasonic bath. Without stirring extraction was carried out through a warm bath at 65 °C. Stirring extraction in an orbital shaker at 65 °C and 150 rpm. The extraction was carried out for a total of 4 h, and sampling was performed hourly for all three methods. The extraction temperature was selected considering the results of previous studies [29,30] in which a higher extraction yield of antioxidants is observed at higher temperatures and the temperature control limitations of the equipment used. Moreover, considering a potential industrial application of the process, it could be more convenient and energetically less costly to work at higher temperatures (65 °C) for shorter times (≤4 h). Then, the extract was filtered through Whatman No.1 filter paper in a funnel to remove nettle residues from the liquid extract. Then, 1/100 dilution of each extract was made, and the diluted extracts were stored in darkness at 5 °C until analyses.

### 3.4. Determination of Antioxidant Properties

The TPC present in the extracts was quantified through the Folin–Ciocalteu method as reported elsewhere [31] but with some modifications, using a spectrophotometer V-670 (Jasco Inc., Tokyo, Japan). Twenty µL of the extract was mixed with 1.58 mL of distilled water and 100 µL of Folin–Ciocalteu reagent. Subsequently, 300 µL of saturated sodium carbonate solution was added, and the absorbance of the solution was measured at 765 nm after maintenance under the appropriate conditions. Each batch was analyzed in triplicate.

The TPC was expressed as mg of gallic acid equivalents (GAE) per 1 g of dried nettle determined by calibration with gallic acid. The calibration equation obtained was TPC (mg ac gallic/g sample) = 1010 Abs_765 nm_ − 85.311 and determination coefficient was R^2^ = 0.9993. 

DPPH^•^ method is described elsewhere [31]. A series of solutions were prepared in test tubes by adding 2 mL of extract and 1.5 mL of DPPH solution. Afterwards, the samples were kept in the darkness and at room temperature for 30 min. The absorbance measurement was performed at 517 nm. A blank sample using methanol was used. Each batch was analyzed in triplicate. The results were expressed as % DPPH^•^.

ABTS^•+^ method was followed as described elsewhere [32], to study the antioxidant capacity of the extracts. First, the ABTS^•+^ reactive solution was prepared the day before and kept in darkness for 16–24 h. Afterwards, 2 mL of extract was mixed with 3 mL of ABTS^•+^ solution and the reaction mixture was incubated for 6 min in darkness at room temperature. The absorbance was measured at 734 nm. The results were expressed as % ABTS^•+^ scavenging activity.

### 3.5. Statistical Analysis

The results obtained were statistically analyzed using Design expert 11^®^ software (Stat-Ease, Minneapolis, MN, USA). Predictive mathematical models were obtained for the optimization of the method. Analysis of variance (ANOVA) was used to statistically validate the model. The variables studied were extraction time expressed as A, extraction method (without stirring, ultrasound, or stirring) expressed as B and the solvent used (methanol, water, or ethanol) expressed as C. The effect of these variables on the antioxidant capacity (TPC, ABTS^•+^, and DPPH^•^) were evaluated. Cook’s distance was used to detect outliers [33]. The interrelationship between extraction time (continuous variable) and the dependent variables (TPC, ABTS^•+^, and DPPH^•^) were established through an equation including linear, interaction, and second-order terms for each combination of extraction method and solvent (categorical variables). The mathematical model used as the first approach was a polynomial model of quadratic order. Partial models of the quadratic model were also fitted and analyzed by ANOVA.

## 4. Conclusions

The results of the process optimization indicated that the most suitable extraction would be using ultrasound treatment with water as solvent and an extraction time of 3.15 h. Moreover, this method reduces the working time and increases the antioxidant activity of the nettle extracts. This work proves that aqueous extract of dried nettle leaves obtained through ultrasound is an important source of natural antioxidants that can be considered as a potential alternative to synthetic additives.

## Figures and Tables

**Table 1:** Experimental design and results for antioxidant variables of dried nettle extracts. TPC is Total Phenolic Content, DPPH^•^ is 1,1-Diphenyl-2-picrylhydrazyl assay and ABTS^•+^ is 2,2-azino-bis(3-etilbenzotiazolin)-6-sulfonic acid assay.

Extraction Time (h)	Method	Solvent	TPC (mg eq Gallic ac/g Dried Nettle)	DPPH^•^ (%)	ABTS^•+^ (%)
1	Without stirring	Methanol	7.3 ± 0.1	75.0 ± 2.1	47.8 ± 1.6
2	Without stirring	Methanol	6.2 ± 0.1	66.6 ± 1.1	39.6 ± 1.1
3	Without stirring	Methanol	8.1 ± 0.4	87.1 ± 1.1	58.4 ± 0.9
4	Without stirring	Methanol	7.3 ± 0.1	73.1 ± 1.3	52.1 ± 0.6
1	Without stirring	Water	11.5 ± 0.1	69.7 ± 1.4	63.7 ± 0.1
2	Without stirring	Water	12.4 ± 0.2	85.4 ± 0.4	82.6 ± 4.9
3	Without stirring	Water	15.3 ± 1.1	84.5 ± 0.8	82.2 ± 2.9
4	Without stirring	Water	13.4 ± 0.6	87.7 ± 0.2	90.8 ± 1.0
1	Without stirring	Ethanol	3.7 ± 0.3	32.9 ± 2.2	27.7 ± 1.7
2	Without stirring	Ethanol	3.4 ± 0.2	33.7 ± 1.7	30.5 ± 0.6
3	Without stirring	Ethanol	3.5 ± 0.2	37.1 ± 3.4	33.0 ± 1.6
4	Without stirring	Ethanol	4.5 ± 0.6	33.6 ± 1.5	34.2 ± 1.3
1	Ultrasound	Methanol	5.5 ± 0.3	69.4 ± 5.5	69.9 ± 5.4
2	Ultrasound	Methanol	10.0 ± 0.6	89.1 ± 0.7	89.3 ± 0.7
3	Ultrasound	Methanol	13.2 ± 0.4	90.2 ± 0.5	90.4 ± 0.5
4	Ultrasound	Methanol	12.5 ± 0.5	89.5 ± 0.3	89.6 ± 0.3
1	Ultrasound	Water	12.8 ± 0.0	50.2 ± 1.4	32.4 ± 0.4
2	Ultrasound	Water	12.1 ± 0.3	78.6 ± 0.7	43.0 ± 0.1
3	Ultrasound	Water	24.2 ± 2.1	82.8 ± 0.6	80.4 ± 1.8
4	Ultrasound	Water	27.0 ± 1.4	85.0 ± 1.0	91.8 ± 2.6
1	Ultrasound	Ethanol	3.6 ± 0.0	29.9 ± 0.3	16.4 ± 2.2
2	Ultrasound	Ethanol	7.5 ± 0.4	56.7 ± 0.8	37.6 ± 0.7
3	Ultrasound	Ethanol	9.3 ± 1.2	65.7 ± 0.5	42.2 ± 0.5
4	Ultrasound	Ethanol	8.2 ± 0.5	52.4 ± 4.4	38.5 ± 0.8
1	Stirring	Methanol	11.1 ± 0.6	69.0 ± 0.5	86.6 ± 0.5
2	Stirring	Methanol	8.6 ± 0.6	57.6 ± 2.2	74.1 ± 2.6
3	Stirring	Methanol	9.1 ± 0.2	54.0 ± 1.7	70.3 ± 0.1
4	Stirring	Methanol	8.8 ± 0.3	65.3 ± 1.2	75.4 ± 4.4
1	Stirring	Water	10.7 ± 0.3	25.8 ± 2.0	47.5 ± 3.8
2	Stirring	Water	10.9 ± 0.6	85.9 ± 0.9	33.8 ± 3.6
3	Stirring	Water	13.3 ± 0.6	91.1 ± 0.6	33.3 ± 1.1
4	Stirring	Water	12.9 ± 0.4	85.9 ± 1.0	21.8 ± 4.5
1	Stirring	Ethanol	7.4 ± 0.2	61.0 ± 0.4	30.4 ± 4.7
2	Stirring	Ethanol	5.2 ± 0.6	48.8 ± 0.2	8.1 ± 0.6
3	Stirring	Ethanol	6.5 ± 0.1	66.5 ± 1.2	16.9 ± 0.9
4	Stirring	Ethanol	5.9 ± 0.4	62.1 ± 0.9	13.4 ± 6.2

**Table 2:** Analysis of variance (ANOVA) and fit statistics for total phenolic content (TPC), %DPPH^•^ (1,1-diphenyl-2-picrylhydrazyl) and %ABTS^•+^ (2,2-azino-bis(3-etilbenzotiazolin)-6-sulfonic acid) results. A refers to time (h), B refers to method (without stirring, ultrasound, or stirring), and C refers to solvent (methanol, water, or ethanol).

Source	TPC		%DPPH^•^		%ABTS^•+^	
	F-Value	*p*-Value	F-Value	*p*-Value	F-Value	*p*-Value
Model	20.85	2.14 × 10^−9^	7.30	3.07 × 10^−5^	24.85	3.78 × 10^−10^
A	20.56	1 × 10^−4^	13.09	1.6 × 10^−3^	8.77	7.1 × 10^−3^
B	16.98	3.46 × 10^−5^	1.22	3.15 × 10^−1^	13.87	1 × 10^−4^
C	80.10	7.94 × 10^−11^	25.55	2.37 × 10^−6^	83.84	5.10 × 10^−11^
AB	14.46	9.75 × 10^−5^	1.17	3.29 × 10^−1^	15.58	6.07 × 10^−5^
AC	5.20	1.41 × 10^−2^	3.58	4.59 × 10^−2^	2.12	1.43 × 10^−1^
BC	4.24	1.06 × 10^−2^	5.25	4.2 × 10^−3^	20.85	3.17 × 10^−7^
A^2^			5.17	3.34 × 10^−2^		
r^2^	0.92		0.82		0.93	
Adjusted r^2^	0.88		0.71		0.89	
Predicted r^2^	0.80		0.44		0.81	
Adequate precision	20.28		9.00		17.94	

**Table 3:** Mathematical models for the prediction of total phenolic content (TPC, mg eq gallic acid/g dried nettle), %DPPH^•^ (1,1-diphenyl-2-picrylhydrazyl) and %ABTS^•+^ (2,2-azino-bis(3-etilbenzotiazolin)-6-sulfonic acid) values as a function of extraction time, for each method and solvent used.

Extraction Method	Solvent	Model
Without stirring	Methanol	
Without stirring	Water	TPC = 9.1 + 1.6 × *Time*(h)
Without stirring	Ethanol	TPC = 4.4 − 0.2 × *Time*(h)
Ultrasound	Methanol	TPC = 3.7 + 2.7 × *Time*(h)
Ultrasound	Water TPC = 7.5 − 0.1 × *Time*(h)	TPC = 8.1 + 4.4 × *Time*(h)
Ultrasound	Ethanol	TPC = 0.8 + 2.5 × *Time*(h)
Stirring	Methanol	TPC = 10.8 − 0.6 × *Time*(h)
Stirring	Water	TPC = 9.0 + 1.2 × *Time*(h)
Stirring	Ethanol	TPC = 8.0 − 0.7 × *Time*(h)
Without stirring	Methanol	DPPH^•^ (%) = 58.6 + 18.8 × *Time*(h) − 4.0 × *Time*^2^ (h)
Without stirring	Water	DPPH^•^ (%) = 41.0 + 28.3 × *Time*(h) − 4.0 × *Time*^2^ (h)
Without stirring	Ethanol	DPPH^•^ (%) = 13.9 + 20.2 × *Time*(h) − 4.0 × *Time*^2^ (h)
Ultrasound	Methanol	DPPH^•^ (%) = 53.1 + 24.6 × *Time*(h) − 4.0 × *Time*^2^ (h)
Ultrasound	Water	DPPH^•^ (%) = 18.9 + 34.2 × *Time*(h) − 4.0 × *Time*^2^ (h)
Ultrasound	Ethanol	DPPH^•^ (%) = 16.3 + 25.9 × *Time*(h) − 4.0 × *Time*^2^ (h)
Stirring	Methanol	DPPH^•^ (%) = 34.6 + 22.7 × *Time*(h) − 4.0 × *Time*^2^ (h)
Stirring	Water	DPPH^•^ (%) = 21.5 + 32.3 × *Time*(h) − 4.0 × *Time*^2^ (h)
Stirring	Ethanol	DPPH^•^ (%) = 29.3 + 24.1 × *Time*(h) − 4.0 × *Time*^2^ (h)
Without stirring	Methanol	ABTS^•+^ (%) = 42.1 + 2.8 × *Time*(h)
Without stirring	Water	ABTS^•+^ (%) = 59.3 + 8.2 × *Time*(h)
Without stirring	Ethanol	ABTS^•+^ (%) = 24.6 + 2.7 × *Time*(h)
Ultrasound	Methanol	ABTS^•+^ (%) = 60.1 + 9.9 × *Time*(h)
Ultrasound	Water	ABTS^•+^ (%) = 23.8 + 15.3 × *Time*(h)
Ultrasound	Ethanol	ABTS^•+^ (%) = 10.0 + 9.8 × *Time*(h)
Stirring	Methanol	ABTS^•+^ (%) = 94.1 − 7.0 × *Time*(h)
Stirring	Water	ABTS^•+^ (%) = 38.2 − 1.6 × *Time*(h)
Stirring	Ethanol	ABTS^•+^ (%) = 34.9 − 7.1 × *Time*(h)

## Data Availability

Data is contained within the article.

## Sample Availability

Not available.
